# Comparison of the Accuracy of Bracket Placement with Height Bracket Positioning Gauge and Boone Gauge

**DOI:** 10.5681/joddd.2011.026

**Published:** 2011-12-19

**Authors:** Amir Mohammadi, Seyed Hossein Moslemzadeh

**Affiliations:** ^1^Assistant Professor, Department of Orthodontics, Faculty of Dentistry, Tabriz University of Medical Sciences, Tabriz, Iran

**Keywords:** Boone gauge, bracket positioning, bracket placement, height bracket positioning gauge

## Abstract

**Background and aims:**

Diverse gauges have been used to measure and determine bracket height for correct bracket positioning. The aim of the present study was to determine and compare bracket positioning accuracy by using height bracket positioning gauge (HBPG) and Boone gauge (BG).

**Materials and methods:**

Nineteen sets of stone models were prepared from one patient. One set was employed to de-termine the ideal position of brackets, and the remaining nine pairs of sets for bracket placement by nine clinicians usingHBPG and BG. Teeth were then sectioned from the stone models and placed inside acrylic molds; photographs were takenand imported to a computer. In two groups, the position of each bonded bracket was compared in three aspects of vertical, mesiodistal and angular with the ideal position of every bracket. Finally, bracket positioning errors were measured.

**Results:**

Mann-Whitney U test demonstrated significant differences in the means ofvertical error between the HBPG group and BG groups (P<0.001), while there were no significant differences between mesiodistal and angular errors. Facto-rial ANOVA revealed that gauge and tooth type, and the position of tooth on the right and left side of the mouth play a ma-jor role in the rate of vertical error.

**Conclusion:**

Vertical accuracy of bracket positioning by the use of HBPG is more than that by BG. However, there is no difference between two gauges in relation to the mesiodistal and angular errors.

## Introduction

Correct bracket placement is necessary to achieve maximum advantages from fixed orthodontic appliances, especially preadjusted ones. This in turn facilitates the final phases of the treatment and leads to an optimal occlusion.^1 - 5^

A gauge is used to measure and determine the bracket distance from the incisor or occlusal edge of the teeth.^2 - 4 , 6 - 9^ In this context, various gauges have been introduced. The most commonly used ones are height bracket positioning gauge (HBPG) and Boone gauge (BG).^10^

According to Angle, the best position of the band is where it fits better mechanically. Therefore, if feasible, the bracket should be placed at the center of the labial surface of the tooth.^11^ Andrews used facial axis of clinical crown (FACC) as a guideline and believed that its middle point is a reliable location to use in straight wire appliance (SWA).^12 - 17^ McLaughlin and Bennett proposed a table to determine vertical heights of brackets. In this method, at first the length of clinical crowns which are completely erupted is measured. Then, a row of this table which has the closest numbers to the obtained measures is selected and brackets are placed in the proper position by means of a gauge. In this method, in addition to the use of clinical crown center, a gauge is used to increase vertical precision.^12 - 14^

Apart from height bracket positioning gauge and Boone gauge (Figure 1), there are other gauges introduced by Droschl, Samuels, and Geron.^10 , 18 - 20^


Figure 1. Boone gauge (a) and height bracket positioning gauge (b).

**a:**
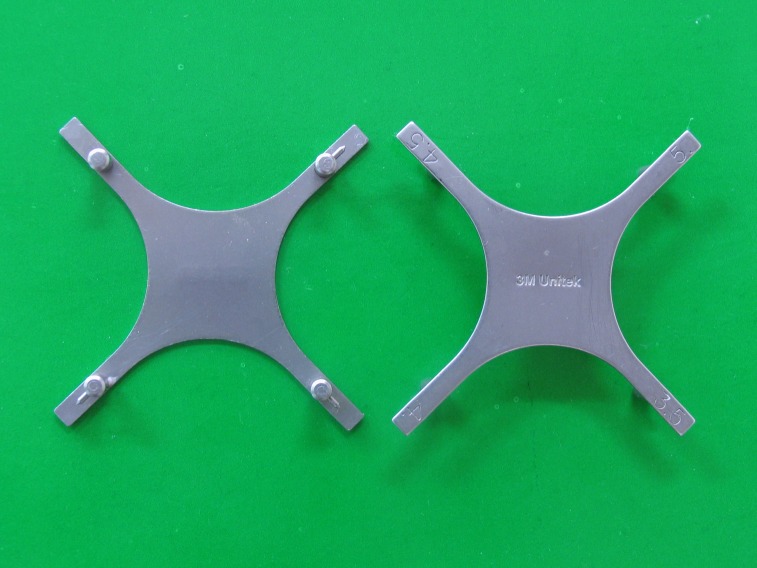

**b:**
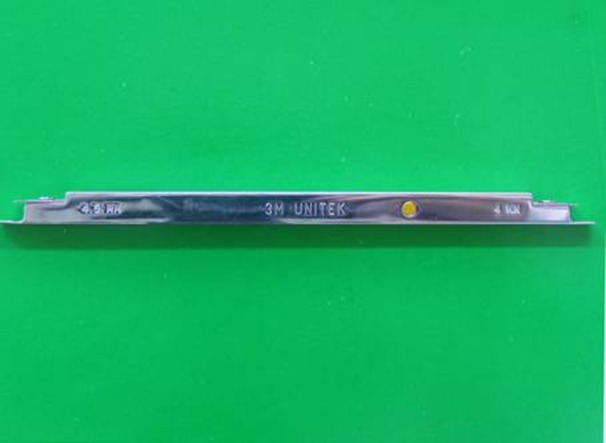

Aguirre et al^21^ conducted a study to compare bracket positioning accuracy. They found that measurement error in angulation is more than vertical and mesiodistal positions. Fowler et al^22^ in their study to measure the accuracy of bracket placement reported the maximum error in determining long axis of clinical crown (LACC) angle and then in the height of the midpoint of crown (LA point), respectively, while they observed the minimal error in mesiodistal LA position. Balut et al^23^ reported average vertical and angular errors of 0.34 mm and 5.54 degrees, respectively, in placing preadjusted brackets. Koo et al^24^ investigated the accuracy of bracket placement between direct and indirect bonding techniques. In a similar study, Hodge et al^25^ did not find any discrepancies in the overall error rates of direct and indirect bracketing despite the fact that there was more error in height than in mesiodistal position and less error in angular position in comparison with other dimensions.Armestong et al^26^ concluded that accurate direct bonding of orthodontic brackets to teeth does not appear to be related to clinical experience or specialist training. Armestong et al,^27^ in another study, compared accuracy of bracket positioning between two techniques, localizing the center of the clinical crown and measuring the distance from the incisal edge. They reported that bracket bonding guided by measuring the distance from incisal edge may result in improved placement for anterior teeth.

Although the accuracy of the bracket placement and the rate of accuracy between direct and indirect bonding techniques have been investigated and compared in the literature, they all have used only one gauge type. To the best of our knowledge no study to date has compared the accuracy of bracket placement using different gauges, and probably gauge type is one of the reasons behind discrepancies in the findings in the literature. In relation to our clinical practice, the accuracy of bracket placement using HBPG and BG might be different.

The aim of the present study was to determine and compare the accuracy of bracket placement in terms of bracket height, mesiodistal position and angulation using HBPG and BG.

## Materials and Methods

A patient with Class II, division 1, malocclusion and mild crowding was selected. The case under investigation had normal and fully erupted teeth and the incisor edge or cusp tip was without any erosion, fracture or previous restoration. The sizes of the teeth were quite normal and the malocclusion did not hinder the ideal positioning of brackets.

The impressions were made with alginate and 19 sets of stone models were provided. One set of stone models was used to determine and draw LACC and mark the desired height while others were divided into two groups: group A consisted of 9 stone model sets in order to position brackets with the use of HBPG (3M/Unitek ^TM ^, Monrovia, CA, USA) and group B included 9 sets of stone models to position brackets with the use of BG (3M/Unitek^TM^ , Monrovia, CA, USA).

The height of the ideal stone model teeth was measured and the height of bracket placement was determined according to the tables provided by McLaughlin and Bennett. To simulate the oral cavity conditions, the models were mounted on a mannequin.

Mini Mono MBT 022 bracket types (Forestadent, Pforzheim, Germany) were used due to their high accuracy of the bracket slot.^28^ With the use of respective gauges for each stone model, brackets from the second premolar on one side to the second premolar on the other side were bonded in the given height using light-cured composite resin [Transbond XT (3M/Unitek^TM ^, Monrovia, CA, USA)] and then exposed to light from each side for 10 seconds by 9 clinicians.

To determine and draw FACC on the ideal group’s teeth, every tooth was examined on all sides by 3 clinicians and eventually the line was drawn in pencil.

The desired height was also marked by means of a digital caliper on FACC. This step was completed after bracketing in two groups to eliminate any biases. All the teeth in all the stone models were sectioned by a special saw and separated from the cast without inflicting any damage on their crown contours.

A digital camera (Canon, model Power Shot Pro 1) was utilized to take photographs. The camera was set at super-macro at a focal distance of f=8.0.

The distance between the camera lens and the tooth was 54 mm. An acrylic mold was used to provide a fixed and repeatable position for taking photographs. To this end, palatal or lingual and occlusal or incisal and mesial and distal surfaces (more occlusal from the contact point) from each of the ideal group’s teeth were placed inside the acrylic mold. With this method the teeth were fixed in a repeatable position. All the four acrylic molds of teeth were put in a tray (Figure 2a). This tray was placed inside a container which was fixed on the camera base telescopically. Then, the teeth were placed in a fixed position at a definite distance from the camera so that the camera lens was situated vertical to FACC at a specific point from the occlusogingival and mesiodistal aspects. Photographs were taken from all the acrylic molds by fixed camera while the distance between the camera and the acrylic molds containing the teeth was kept constant (Figure 2b).


Figure 2. Acrylic molds of the teeth were placed in a tray (a). Digital camera for taking photographs (b).

**a:**
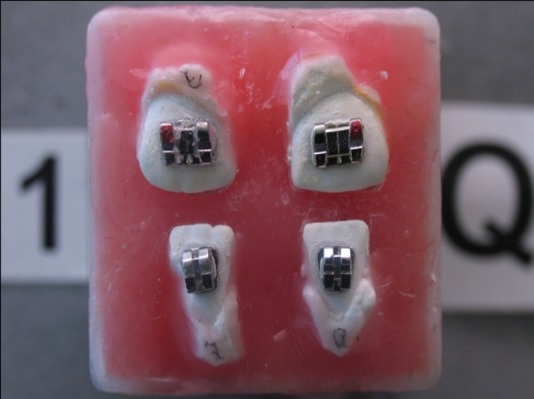

**b:**
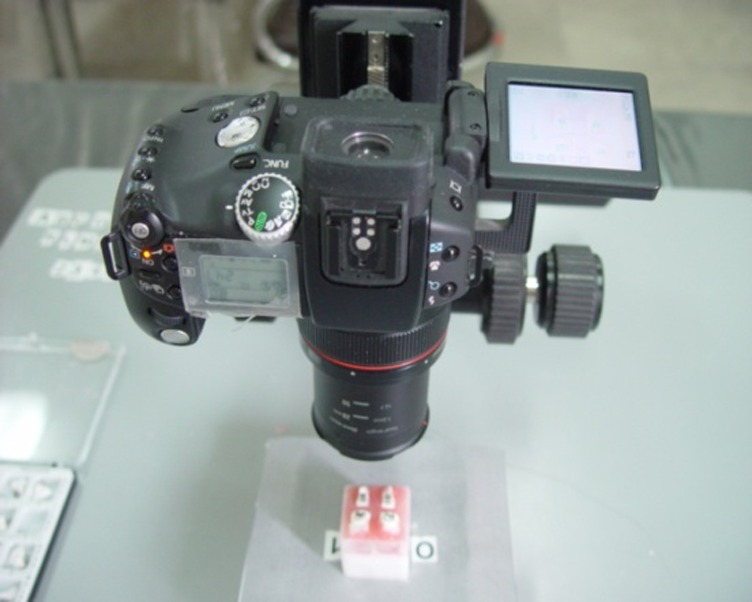

The photographs were then imported to a computer and tooth outlines, FACC lines and the marked heights were drawn for all the teeth in the ideal group by the use of CorelDraw Software V13, and the results were saved in Windows Meta File (WMF) formats. In both groups, the vertical and horizontal distances of the two bracket wings were measured by the computer and then central vertical lines and central bracket points were drawn.

**Figure 3:**
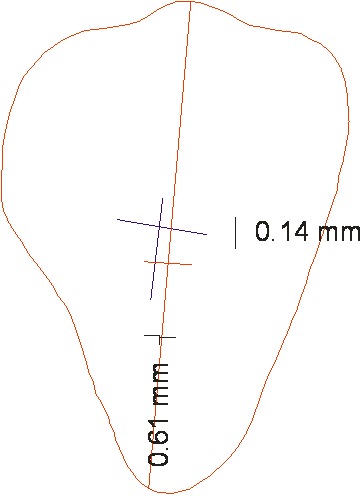
Superimposition of the ideal tooth and experimental groups: tracing of crown outline, facial axis of clinical crown—FACC—and LA point in the ideal group (red line). The vertical and horizontal lines were drawn with the same distance of the two wings of the bracket in the experimental groups (black line), and their differences were measured in vertical and horizontal dimension.

The drawn outline of the ideal cast was superimposed on similar teeth in the two groups. Two distances and one angle were measured between the experimental and ideal groups (Figure 3). The marked point on FACC in the ideal cast was considered zero point. If the bracket was positioned gingival or mesial to the ideal, the value was considered “+”, and “−” meaning the bracket was occlusal or distal to the ideal. In relation to angulation error, if occlusal central vertical line of the experimental bracket was more mesial to the ideal, it was defined as “+” and the opposite was defined as “−”. During photography, a linear index with a definite length was also used. Therefore, magnification rate in photography was calculated.

### Statistics and data analysis

Data were analyzed descriptively on the basis of original and absolute values. Kolmogorov-Smirnov test was used to analyze normal distribution of data and Mann-Whitney U test was applied to determine the difference in means between the two groups and finally the effects of the major variables were investigated with the use of factorial ANOVA.

## Results

In this study, original values were used. The error rates in the three dimensions for each specific gauge is represented in Table 1.

**Table 1:** Error rates in three dimensions for each specific gauge with the use of original values

	Height bracket positioning gauge						Boone gauge					
Error	N	Min	Max	Mean	SD	CV	N	Min	Max	Mean	SD	CV
Vertical (mm)	180	−1.75	0.90	−0.06	0.49	−8.16	180	−1.57	1.02	−0.39	0.49	−1.25
Horizontal (mm)	180	−1.89	1.12	−0.28	0.45	−1.60	180	−1.44	0.74	−0.29	0.44	−1.51
Angular (−)	180	−10.96	15.66	0.21	4.68	22.2	180	−9.73	12.82	−0.09	4.69	−52.11

SD: standard deviation; CV: coefficient of variant.

Kolmogorov-Smirnov test showed normal distribution of data. Original values were used to examine the discrepancy in the error rates for each tooth in the two groups. Despite the fact that both groups had normal distribution, non-parametric Mann-Whitney U test was used due to the limited number of samples (9 teeth in each group). The results were reported separately for each aspect of the study.

### Measurement of vertical error in bracket positioning

Figures 4a and 4b show the vertical error distributions and ranges of bracket positioning in the two distinct groups in relation to the ideal position. Table 2 also illustrates the vertical error means for each tooth by two gauges separately. The overall mean vertical error with the use of HBPG was −0.06 mm while it was −0.39 mm with the use of BG, which is statistically significant (p<0.001). However, this difference was not statistically significant in all the teeth, i.e. the brackets were more occlusal when BG was used.

**Table 2:** The vertical error means for each tooth with the use of the two gauges separately

Tooth	Height bracket positioning gauge			Boone gauge			Mann−Whitney U	P
	Number	Mean Rank	Mean	Number	Mean Rank	Mean		
UR_1_	9	11.83	0.17	9	7.17	−0.19	19.5	0.063
UL_1_	9	13.22	0.38	9	5.78	−0.27	7.0	0.003*
LR_1_	9	12.33	−0.04	9	6.67	−0.44	15.0	0.024*
LL_1_	9	13.33	−0.08	9	5.67	−0.47	6.0	0.002*
UR_2_	9	12.33	0.01	9	6.67	−0.42	15.0	0.024*
UL_2_	9	12.11	0.33	9	6.89	0.03	17.0	0.038*
LR_2_	9	12.44	−0.27	9	6.56	−0.66	14.0	0.019*
LL_2_	9	10.94	0.28	9	8.06	0.15	27.0	0.251
UR_3_	9	9.78	0.32	9	9.22	0.30	38.0	0.825
UL_3_	9	12.89	0.30	9	6.11	0.00	10.0	0.007*
LR_3_	9	11.39	−0.11	9	7.61	−0.32	23.5	0.132
LL_3_	9	9.78	0.06	9	9.22	0.01	38.0	0.825
UR_4_	9	11.61	−0.15	9	7.39	−0.37	21.5	0.093
UL_4_	9	12.33	0.00	9	6.67	−0.36	15.0	0.024*
LR_4_	9	9.78	−0.78	9	9.22	−0.96	38.0	0.825
LL_4_	9	10.61	−0.65	9	8.39	−0.84	30.5	0.377
UR_5_	9	11.44	−0.33	9	7.56	−0.54	23.0	0.122
UL_5_	9	11.78	0.09	9	7.22	−0.34	20.0	0.070
LR_5_	9	11.89	−0.68	9	7.11	−1.10	19.0	0.058
LL_5_	9	13.22	−0.24	9	5.78	−0.96	7.0	0.003*
Total	180	216.06	−0.06	180	144.94	−0.39	9798.5	0.000*

UR: upper right; UL: upper left; LR: lower right; LL: lower left; numbers indicate the number of teeth in the Palmer system. * Statistically significant (P <0.05).

###  Measurement of mesiodistal error in bracket positioning

Figures 4c and 4d indicate the horizontal error distributions and ranges of bracket positioning in the two distinct groups in relation to the ideal position. A comparison of the two groups revealed that the difference in mesiodistal error with the use of gauge was not statistically significant. The overall mean mesiodistal error with the use of HBPG was −0.28 mm while it was −0.29 mm with the use of BG, with no statistically significant differences (p=0.982).

### Measurement of angular error in bracket positioning

The angular error distribution and range of bracket positioning in the two distinct groups in relation to the ideal position are represented in Figures 4c and 4d. The findings show a significant difference between the two groups only in the upper right canine (p=0.047): the occlusal part of the central line in the bracket was more mesial (mesial tip) when HBPG was used. However, this discrepancy was not significant in the other teeth. The overall mean angular error with the use of HBPG was 0.21 degrees while it was −0.09 degree with the use of BG, with no statistically significant differences (p=0.914).


Figure 4. The range of bracket positioning and distribution of vertical error (a), mesiodistal error (b), and angular error (c) in the HBPG group in relation to the ideal position. The range of bracket positioning and distribution of vertical error (d), mesiodistal error (e), and angular error (f) in the BG group in relation to the ideal position. UR: upper right; UL: upper left; LR: lower right; LL: lower left; numbers indicate the number of teeth in the Palmer system.

**a:**
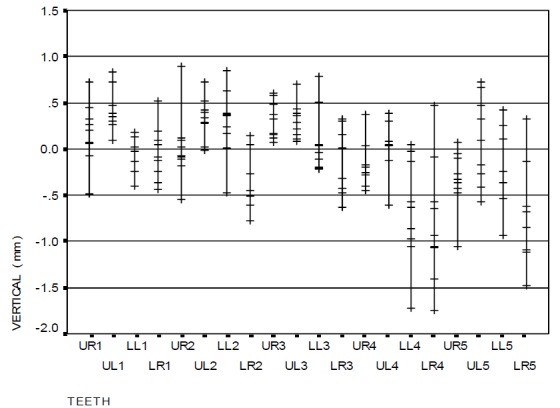

**b:**
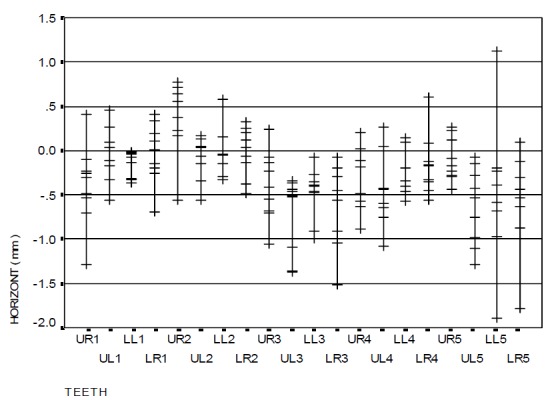

**c:**
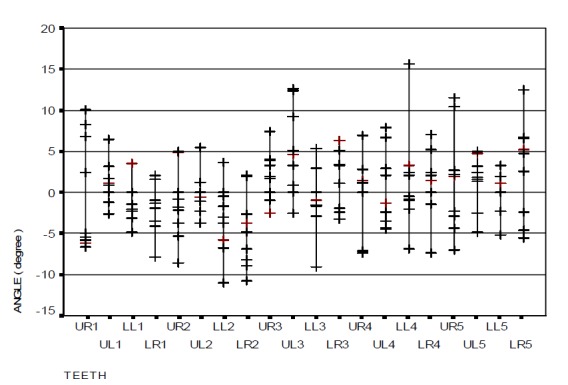

**d:**
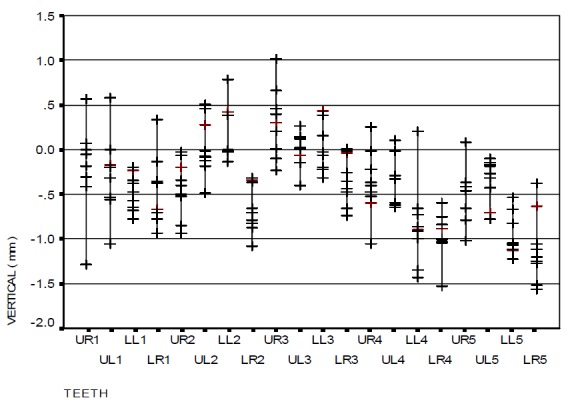

**e:**
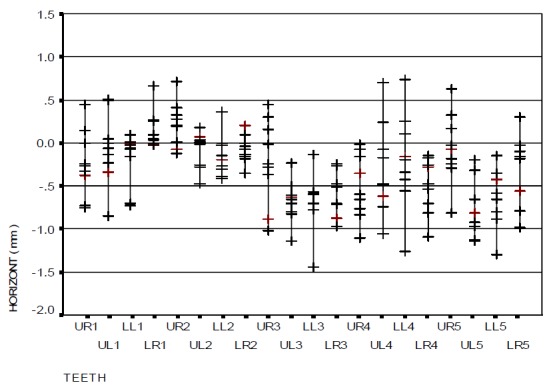

**f:**
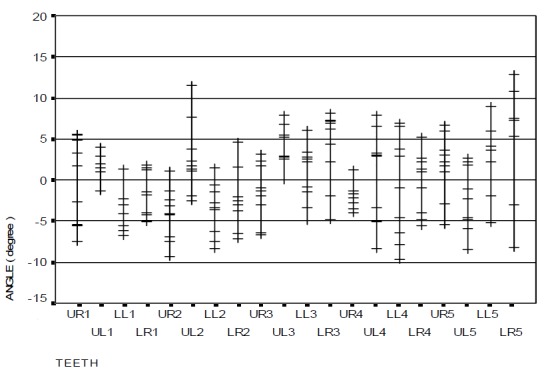

### Effective variables in vertical error in bracket positioning

Since there was a significant difference between the two gauges in the vertical error of bracket placement, univariate ANOVA was employed to compare the effects of the major variables. Table 3 shows that the effects of gauge and tooth types, tooth location in the upper or lower jaws or on the right or left side of the mouth were significant (p<0.001). Moreover, when the two variables are taken into account and one of them is tooth, the differences in the vertical errors are significant (p<0.05). In other words, the vertical error in bracket placement is different in different teeth.

**Table 3:** Effective variables in the vertical error in bracket positioning

Source	Type III Sum of Squares	df	Mean square	F	P value
Corrected Model	55.239(a)	39	1.416	11.162	.000*
Intercept	19.035	1	19.035	150.001	.000*
Gauge	9.274	1	9.274	73.080	.000*
Jaw	11.435	1	11.435	90.110	.000*
Side	3.680	1	3.680	29.003	.000*
Tooth	21.184	4	5.296	41.734	.000*
Gauge – Jaw	.013	1	.013	.102	.749
Gauge – Side	.101	1	.101	.799	.372
Jaw – Side	.345	1	.345	2.717	.100
Gauge – Jaw – Side	.193	1	.193	1.523	.218
Gauge – Tooth	1.291	4	.323	2.542	.040*
Jaw – Tooth	2.393	4	.598	4.715	.001*
Gauge – Jaw – Tooth	.429	4	.107	.845	.498
Side – Tooth	3.332	4	.833	6.565	.000*
Gauge – Side – Tooth	.509	4	.127	1.003	.406
Jaw – Side – Tooth	.894	4	.224	1.761	.136
Gauge – Jaw – Side – Tooth	.166	4	.042	.327	.860
Error	40.607	320	.127	–	–
Total	114.881	360	–	–	–
Corrected Total	95.847	359	–	–	–

As tested by ANOVA. * Statistically significant (P <0.05).

## Discussion

In previous studies, either original or absolute values have been used and the statistical analyses and the interpretation of the results have all been based on one of them. This procedure has resulted in huge discrepancies in the results. In this study, original values were used to demonstrate the rate, direction, distribution, range and other features of bracket positioning error in order to meet the objectives.

Regardless of the gauge, the overall means of in bracket positioning errors with original values in vertical aspect were −0.22 mm and −0.29 mm in mesiodistal and 0.15 degree in angulation, respectively. In addition, through absolute values, the overall means of bracket positioning errors in the vertical aspect were 0.43 mm were 0.41 mm in the mesiodistal and 3.76 degrees in angulation.

In a study by Fowler et al^22^ the overall vertical and mesiodistal errors were ±0.32 mm and ±0.20 mm, respectively. The minimum angular error was ±2.61 degrees and the maximum error was ±3.75 degrees. Given the fact that in their study only LACC and LA point were determined, there was no bracket positioning and the stone models were at the disposal of the clinicians freely similar to indirect bracket bonding, i.e. they were not mounted on the mannequin and the low error rate was expected and predictable. In a study by Balut et al^23^ vertical error rate was 0.34 mm and angular error rate was 5.56 degrees. In comparison, their vertical error was less and angular error was more than the findings in this study.

In addition, in a study by Koo et al^24^ using absolute values, the overall vertical error mean for bracket positioning was 0.35 mm and the mesiodistal and angular error means were 0.19 mm and 2.57 degrees, respectively. Considering the fact that Boone gauge was used in their study, it is necessary to utilize the same measures to compare the findings in both studies (Table 3). The comparison of the results led us to the conclusion that the mesiodistal error in a study carried out by Koo et al was much lower than that in the present study. Such discrepancy might be attributed to differences in photography techniques.

In a study by Hodge et al^25^ the vertical, mesiodistal, and angular error means were found to be 0.27 mm (gingivally), −0.11 mm (distally), and 0.08, respectively. However, in the present study, the findings with the use of HBPG were −0.06 mm (occlusally), −0.28 mm (distally), and 0.21 degree.

Based on the findings of this study, it can be concluded that a remarkable error occurs in all the three vertical, mesiodistal and angular aspects with the use of both gauges. However, there is a significant difference in terms of vertical error between the two gauges. Thus, because of the imprecision in bracket positioning with HBPG and BG gauges, it is not possible to reach the ideal tooth positions with the application of preadjusted brackets and straight wire concept without implementing compensatory bends on the wire.

## Conclusion

Using two gauges, namely height bracket positioning gauge (HBPG) and Boone gauge (BG), this study investigated the accuracy of bracket positioning in vertical, mesiodistal, and angular aspects. The following conclusions can be drawn based on this study:

1. The use of HBPG gauge results in less vertical error and better accuracy in bracket positioning in comparison to BG (p<0.001), with no significant differences between the two gauges in terms of mesiodistal and angular accuracy.
2. In general, height bracket positioning gauge (HBPG) is recommended.
